# Molecular Characterization of Invasive Meningococcal Isolates from Countries in the African Meningitis Belt before Introduction of a Serogroup A Conjugate Vaccine

**DOI:** 10.1371/journal.pone.0046019

**Published:** 2012-09-27

**Authors:** Dominique A. Caugant, Paul A. Kristiansen, Xin Wang, Leonard W. Mayer, Muhamed-Kheir Taha, Rasmata Ouédraogo, Denis Kandolo, Flabou Bougoudogo, Samba Sow, Laurence Bonte

**Affiliations:** 1 WHO Collaborating Centre for Reference and Research on Meningococci, Norwegian Institute of Public Health, Oslo, Norway; 2 Faculty of Medicine, University of Oslo, Oslo, Norway; 3 WHO Collaborating Center for Prevention and Control of Epidemic Meningitis, Centers for Disease Control and Prevention, Atlanta, Georgia, United States of America; 4 WHO Collaborating Centre for Reference and Research on Meningococci, Institut Pasteur, Paris, France; 5 Laboratoire de Réference Méningite, Centre Hospitalier Universitaire Pédiatrique Charles de Gaulle, Ouagadougou, Burkina Faso; 6 WHO Inter country Support Team for West Africa, Ouagadougou, Burkina Faso; 7 Institut National de Recherche en Santé Publique (INRSP), Bamako, Mali; 8 Centre pour les Vaccins en Développement (CVD), Bamako, Mali; 9 Support Logistique, Médecins Sans Frontières, Paris, France; Health Protection Agency, United Kingdom

## Abstract

**Background:**

The serogroup A conjugate meningococcal vaccine, MenAfriVac, was introduced in mass vaccination campaigns in December 2010 in Burkina Faso, Mali and Niger. In the coming years, vaccination will be extended to other African countries at risk of epidemics. To document the molecular characteristics of disease-causing meningococcal strains circulating in the meningitis belt of Africa before vaccine introduction, the World Health Organization Collaborating Centers on Meningococci in Europe and United States established a common strain collection of 773 isolates from cases of invasive meningococcal disease collected between 2004 and 2010 from 13 sub-Saharan countries.

**Methodology:**

All isolates were characterized by multilocus sequence typing, and 487 (62%) were also analyzed for genetic variation in the surface antigens PorA and FetA. Antibiotic susceptibility was tested for part of the collection.

**Principal Findings:**

Only 19 sequence types (STs) belonging to 6 clonal complexes were revealed. ST-5 clonal complex dominated with 578 (74.8%) isolates. All ST-5 complex isolates were remarkably homogeneous in their PorA (P1.20,9) and FetA (F3-1) and characterized the serogroup A strains which have been responsible for most epidemics during this time period. Sixty-eight (8.8%) of the 773 isolates belonged to the ST-11 clonal complex which was mainly represented by serogroup W135, while an additional 38 (4.9%) W135 isolates belonged to the ST-175 complex. Forty-eight (6.2%) serogroup X isolates from West Africa belonged to the ST-181 complex, while serogroup X cases in Kenya and Uganda were caused by an unrelated clone, ST-5403. Serogroup X, ST-181, emerged in Burkina Faso before vaccine introduction.

**Conclusions:**

In the seven years preceding introduction of a new serogroup A conjugate vaccine, serogroup A of the ST-5 clonal complex was identified as the predominant disease-causing strain.

## Introduction

The meningitis belt of Africa was first described by Lapeysonnie in 1963 [1] and was further defined by Molesworth and co-workers in 2002 [2]. It now encompasses parts or the whole of 25 countries south of the Sahara, stretching from Ethiopia in the east to Senegal in the west. Every year during the dry season the region is affected by outbreaks of meningococcal meningitis and large epidemics emerge unpredictably 8 to 10 years apart. Traditionally, serogroup A meningococci have been responsible for most of the endemic disease as well as for the large epidemics, but more recently serogroup W135 and serogroup X strains have also been involved [3]–[13]. Emergence and dissemination of a new strain may lead to epidemics under certain environmental conditions. New strains that appeared and were disseminated during the annual Hajj pilgrimage have been linked to several epidemics [14].

The most severe epidemic of meningococcal meningitis experienced by Africa was in 1996, with more than 150,000 reported cases and 16,000 deaths [15]–[17]. It was caused by a serogroup A clone of *Neisseria meningitidis* assigned to sequence type (ST) -5 using multilocus sequence typing [18]. In 2002, Burkina Faso was the first country to experience a major serogroup W135 epidemic, with 13,000 reported cases, and 1,400 deaths [10]. A hypervirulent ST-11 clone was responsible for this epidemic [7].

Preventive vaccination with meningococcal polysaccharide vaccines was usually not attempted because of their relatively short protection and low immunogenicity in young children [19]. Thus, the World Health Organization (WHO) recommended reactive mass vaccination to halt epidemics using either the A/C or the A/C/W135 polysaccharide vaccines, depending on the serogroup of the outbreak strains [20].

Starting in 2002, the Meningitis Vaccine Project (MVP), a public-private partnership between WHO and the Program for Appropriate Technology in Health (PATH), developed an effective monovalent serogroup A conjugate meningococcal vaccine, MenAfriVac, at a price affordable for African countries, with the aim of eliminating the devastating serogroup A epidemics occurring in sub-Saharan Africa [21]. The vaccine was introduced in a country-wide mass vaccination campaign in Burkina Faso in December 2010, where the vaccine was offered to all individuals aged 1 to 29 years. Vaccination of the same age group started in Mali and Niger in 2010 and was completed in 2011 [22].

In 2005 the two WHO Collaborating Centers for Reference and Research on Meningococci in Marseille, France, and Oslo, Norway, published an overview on the phenotypic and genotypic features of meningococcal isolates recovered from meningitis cases between 1988 and 2003 in 13 countries of the African meningitis belt [18]. We report here the molecular characteristics of the strains circulating in these countries from 2004 to 2010, prior to MenAfriVac introduction. The strain collection established by these two WHO Collaborating Centers was supplemented with isolates received by the WHO Collaborating Center for Prevention and Control of Epidemic Meningitis, Centers for Disease Control and Prevention, Atlanta, GA, and by the Institut Pasteur, Paris, France, which is a newly nominated WHO Collaborating Center, following closure of the WHO Center in Marseille in 2010.

## Materials and Methods

### Bacterial isolates

The strain collection comprised a total of 773 isolates, all recovered from cerebrospinal fluid samples from patients of countries of the African meningitis belt and forwarded to the WHO Collaborating Centres. Strains from patients in other African countries were excluded, as were isolates from asymptomatic carriers. The sources of the isolates were as follows: 1) a total of 372 isolates from 2004 to 2009 constituted a large part (88%) of the collection of invasive isolates from the WHO Collaborating Centre in Marseille for that period; the isolates were transferred to the other WHO Collaborating Centres under contract for scientific purposes; 2) 360 isolates were from the WHO Collaborating Centre in Oslo. These were assembled either by physicians working for Médecins Sans Frontières, using Trans-Isolate media [23] to collect samples in areas where meningitis outbreaks were occurring; during field evaluations performed by the WHO Inter-country Support Team, Ouagadougou; or directly by the co-authors; 3) the remaining isolates were sent to the WHO Collaborating Centre by co-authors. Bacterial identification was determined by Gram staining, the oxidase reaction, and standard biochemical tests. The strains were stored at −80°C in brain heart broth with 15% sterile glycerol or in Greaves solution [24].

### Serogrouping

*N. meningitidis* strains were serogrouped by slide agglutination with sera manufactured by the Institut de Médecine Tropicale du Service de Santé des Armées, Marseilles, France, or commercial antisera (Remel, GA, USA).

### Antimicrobial sensitivity testing

Antimicrobial susceptibility testing was performed by determination of the minimal inhibitory concentrations (MIC) using Etest (AB Biodisk, Solna, Sweden). Isolates were tested for susceptibility to penicillin G, amoxicillin, ceftriaxone, ciprofloxacin, chloramphenicol, rifampin, tetracycline and sulphonamides, and classified using the breakpoints from the European Committee on Antimicrobial Susceptibility Testing (http://www.eucast.org/).

### Genotypic characterization

DNA from each strain was prepared by suspending bacteria in Tris-EDTA buffer (10 mM Tris-HCl and 1 mM EDTA), pH 8.0, heating at 95°C for 10 min, and followed by centrifugation at 16,000× *g* for 5 min. The supernatant was used as DNA template for PCR. After DNA amplification by PCR, sequence of fragments from the seven housekeeping genes, *abcZ* (putative ABC transporter), *adk* (adenylate kinase), *aroE* (shikimate dehydrogenase), *fumC* (fumarase), *gdh* (glucose-6-phosphate dehydrogenase), *pdhC* (pyruvate dehydrogenase subunit), and *pgm* (phosphoglucomutase), were analysed on an AB Prism 373, AB Prism 377 or AB 3130XL DNA sequencer (Applied Biosystems, Foster City, CA), according to the method on the MLST website (http://pubmlst.org/neisseria/). The DNA sequences were compared with the existing alleles on the MLST website using sequence query or the meningococcal genome informatics platform (MGIP) for determination of the allele numbers, STs, and clonal complexes of the isolates [25].

Variation in the *porA* and *fetA* genes, coding for the outer membrane proteins PorA and FetA, respectively, was determined by DNA sequencing, as described previously [26], [27]. New MLST alleles and STs were submitted to the MLST database (http://pubmlst.org/neisseria/), together with the strain serogroup and *porA* and *fetA* sequences.

PCR analysis of the genes coding for the polysaccharide capsule was performed for genogroup determination of non-serogroupable isolates as described [28].

### Data analyses

All the data were entered into an Excel database (Microsoft Corporation, Redmond, WA) and analysed using R version 2.10.0 [29].

## Results

### Origin of isolates

The isolates analysed by MLST originated from Benin (n = 12), Burkina Faso (n = 285), Cameroon (n = 24), Central African Republic (CAR) (n = 5), Chad (n = 53), Ghana (n = 6), Kenya (n = 3), Mali (n = 132), Niger (n = 124), Nigeria (n = 57), Sudan (n = 22), Togo (n = 29) and Uganda (n = 21). The number of isolates retrieved by country and year varied greatly, ranging from zero for some countries in some years to 128 from Burkina Faso in 2006 (Table 1). For Burkina Faso, Chad, Mali and Niger, meningococcal isolates were obtained and characterized for at least 6 of the 7 years of the period analysed.

**Table 1:** Serogroup and sequence type (ST) of invasive meningococcal isolates from sub-Saharan Africa in 2004–2010.

Year	Serogroup	Clonal complex	ST	No. of strains (yearly %)	Country source (no. of isolates)
2004	A	5	7	23 (32.4)	Benin (1); CAR^c^ (4); Ghana (4); Niger (13); Nigeria (1)
	A	5	2859	17 (23.9)	Burkina Faso (16); Mali (1);
	W135	11	11	11 (15.5)	Benin (1); Burkina Faso (8); Ghana (2)
	W135	23	4375	1 (1.4)	Burkina Faso (1);
	W135	175	2881	3 (4.2)	Benin (1); Chad (1); Nigeria (1)
	X	181	181	3 (4.2)	Niger (3)
	Y	11	11	1 (1.4)	Burkina Faso (1)
	Y	23	4375	1 (1.4)	Burkina Faso (1)
	Y	167	767	1 (1.4)	Benin (1)
	NG^a^	5	2859	7 (9.9)	Burkina Faso (6); Mali (1)
	NG	23	4375	2 (2.8)	Burkina Faso (2)
	NG	UA^b^	192	1 (1.4)	Burkina Faso (1)
2005	W135	11	11	6 (35.3)	Chad (4); Kenya (2)
	W135	175	2881	4 (23.5)	Niger (4)
	X	181	181	7 (41.2)	Niger (7)
2006	A	5	7	36 (16.0)	Burkina Faso (1); Mali (1); Niger (25); Nigeria (3); Sudan (6)
	A	5	2859	126 (56.0)	Burkina Faso (122); Mali (4)
	A	5	5788	1 (0.4)	Niger (1)
	W135	11	11	14 (6.2)	Chad (1); Mali (5); Sudan (3); Uganda (5)
	W135	11	5779	1 (0.4)	Burkina Faso (1)
	W135	175	2881	5 (2.2)	Benin (3); Niger (2)
	X	181	181	23 (10.2)	Niger (23)
	X	181	5789	1 (0.4)	Niger (2)
	X	UA	5403	9 (4.0)	Kenya (1); Uganda (8)
	Y	167	2953	1 (0.4)	Benin (1)
	Y	23	4375	5 (2.2)	Burkina Faso (4); Niger (1)
	Y	167	767	2 (0.9)	Mali (2)
2007	A	5	7	34 (18.6)	Chad (4); Mali (3); Niger (12); Nigeria (3); Sudan (12)
	A	5	2859	113 (61.7)	Burkina Faso (71); Mali (23); Togo (19)
	W135	11	11	8 (4.4)	Mali (8)
	W135	175	2881	8 (4.4)	Benin (1); Chad (1); Togo (6)
	X	181	181	3 (1.6)	Burkina Faso (3)
	X	UA	5403	6 (3.3)	Uganda (6)
	Y	23	4375	1 (0.5)	Burkina Faso (1)
	Y	167	767	4 (2.2)	Mali (4)
	Y	167	2880	1 (0.5)	Burkina Faso (1)
	Y	167	8620	1 (0.5)	Mali (1)
	Y	UA	192	1 (0.5)	Mali (1)
	NG	5	2859	2 (1.1)	Burkina Faso (1); Mali (1)
	NG	UA	192	1 (0.5)	Burkina Faso (1)
2008	A	5	7	18 (16.8)	Mali (3); Niger (7); Nigeria (7); Sudan (1)
	A	5	2859	62 (57.9)	Burkina Faso (24); Mali (36); Niger (2)
	A	5	6968	1 (0.9)	Burkina Faso (1)
	W135	11	11	5 (4.7)	Cameroon (1); CAR (1); Chad (3)
	W135	175	2881	13 (12.1)	Benin (1); Burkina Faso (1); Cameroon (9); Togo (2)
	X	181	181	3 (2.8)	Benin (1); Togo (2)
	Y	23	4375	1 (0.9)	Burkina Faso (1)
	Y	167	767	2 (1.9)	Benin (1); Mali (1)
	NG	5	2859	1 (0.9)	Mali (1)
	NG	11	11	1 (0.9)	Chad (1)
2009	A	5	7	60 (65.2)	Cameroon (1); Chad (1); Niger (21); Nigeria (37)
	A	5	2859	16 (17.4)	Burkina Faso (6); Mali (9); Niger (1)
	W135	11	11	12 (13.0)	Cameroon (6); Chad (3); Mali (1); Nigeria (2)
	W135	175	2881	4 (4.3)	Cameroon (4)
2010	A	5	7	36 (46.2)	Cameroon (1); Chad (29); Mali (3); Nigeria (1); Uganda (2)
	A	5	2859	24 (30.8)	Burkina Faso (2); Mali (22)
	A	5	8639	1 (1.3)	Burkina Faso (1)
	W135	11	11	8 810.3)	Cameroon (2); Chad (5); Nigeria (1)
	W135	11	8637	1 (1.3)	Nigeria (1)
	W135	175	8638	1 (1.3)	Burkina Faso (1)
	X	181	181	7 (9.0)	Burkina Faso (7)

a NG, Non-groupable as determined by slide agglutination method.

b UA, Unassigned to any clonal complex.

c CAR, Central African Republic.

### Serogroups

Of the 773 isolates, 568 (73.5%) were serogroup A, 105 (13.6%) serogroup W135, 63 (8.2%) serogroup X, 22 (2.8%) serogroup Y and 15 (1.9%) were non-groupable (NG). Serogroup W135 was present in all these 13 countries within the meningitis belt, while serogroup A was recovered from all countries except Kenya. Serogroup X was mainly found in Niger, Burkina Faso and Uganda. Burkina Faso was the only country where all the serogroups were isolated. No serogroup B or C isolate was recovered from any of the countries.

Genogrouping was performed on the 11 of the 15 NG isolates that were still available. The genes for serogroup A and serogroup Y capsule were detected in 5 and 2 isolates, respectively, but no PCR product was obtained with any of the primer sets for the remaining 4 isolates.

### Antibiotic susceptibility

Antimicrobial susceptibility testing was performed for a selection of isolates (Table 2). All isolates tested were susceptible to amoxicillin, ceftriaxone, ciprofloxacin, chloramphenicol and rifampin. Of the 336 isolates tested against sulphonamide, all but 9 were resistant; the exceptions were 8 serogroup X isolates from Burkina Faso and a serogroup W135 isolate from Kenya. Among the isolates tested against tetracycline, all serogroup A strains were resistant while all the serogroup W135 and X isolates were susceptible. Reduced susceptibility to penicillin was seen for 9% of the isolates, but there was no association with the serogroup of the strains.

**Table 2:** Antibiotic susceptibility of invasive meningococcal isolates from sub-Saharan Africa in 2004–2010.

			% of isolates		
Antibiotic	No. of isolates tested	MIC range (µg/ml)	Susceptible	Intermediate	Resistant
Penicillin G	526	0.012–0.64	91	7	2
Amoxicillin	189	0.012–0.19	100	0	0
Ceftriaxone	364	0.001–0.004	100	0	0
Ciprofloxacin	336	0.003–0.008	100	0	0
Chloramphenicol	526	0.38–2	100	0	0
Rifampin	508	0.002–0.25	100	0	0
Tetracycline	169	0.125–8	20	0	80
Sulphonamide	336	0.025–≥1024	2	0	98

### Molecular characterisation

The ST-5 clonal complex dominated the strain collection with 578 isolates (74.8%), followed by 68 (8.8%) isolates of ST-11 complex, 48 (6.2%) isolates of ST-181 complex, 38 (4.9%) isolates of ST-175 complex, 12 (1.6%) isolates of ST-23 complex and 11 (1.4%) isolates of ST-167 complex (Table 3). The remaining 18 isolates belong to a ST that cannot be assigned to any ST complex; ST-192 (3 isolates) and ST-5403 (15 isolates).

**Table 3:** Distribution of lineages found among 773 *N. meningitidis* strains isolated in the African meningitis belt between 2004 and 2010.

Clonal complex	ST	Serogroup	No. (%) of isolates
ST-5	7	A	207 (26.8%)
	2859	A	358 (46.3%)
		NG^a^	10 (1.3%)
	5788	A	1 (0.13%)
	6968	A	1 (0.13%)
	8639	A	1 (0.13%)
ST-11	11	NG	1 (0.13%)
		W135	64 (8.28%)
		Y	1 (0.13%)
	5779	W135	1 (0.13%)
	8637	W135	1 (0.13%)
ST-23	2953	Y	1 (0.13%)
	4375	NG	2 (0.26%)
		W135	1 (0.13%)
		Y	8 (1.04%)
ST-167	767	Y	9 (1.17%)
	2880	Y	1 (0.13%)
	8620	Y	1 (0.13%)
ST-175	2881	W135	37 (4.79%)
	8638	W135	1 (0.13%)
ST-181	181	X	46 (5.95%)
	5789	X	2 (0.26%)
UA^b^	192	NG	2 (0.26%)
		Y	1 (0.13%)
	5403	X	15 (1.9%)

a NG, Non-groupable as determined by slide agglutination method.

b UA, Unassigned to any clonal complex.

The dominating ST-5 complex was composed of 368 (63.7%) ST-2859 isolates, 207 (35.8%) ST-7 isolates, as well as one isolate (0.2%) of each of the STs 6968, 5788 and 8639. The isolates of the ST-5 complex were either serogroup A (568 isolates) or NG (10 isolates) and a total of 344 ST-5 complex isolates that were further subtyped had identical PorA (P1.20,9) and FetA (F3-1). All serogroup A isolates belonged to the ST-5 complex.

The ST-11 clonal complex included 66 (97%) ST-11 isolates and two SLVs of ST-11, ST-5779 and ST-8637, both with one isolate each. The ST-11 complex was mainly composed of serogroup W135 isolates (97%). All 53 W135 ST-11 complex isolates for which PorA was sequenced were P1.5,2. FetA variant F1-1 dominated within the ST-11 complex with 92.7% of sequenced isolates.

The other clonal complex associated with serogroup W135 was the ST-175 complex, with 37 isolates (97.4%) of ST-2881 and one isolate of ST-8638, a SLV of ST-2881 identified in Burkina Faso in 2010. The dominant PorA-FetA combination among the 20 sequenced ST-175 complex isolates was P1.5-1,2-36/F5-1 (85%).

The ST-181 clonal complex consisted of ST-181 (95.8%) and ST-5789 (4.2%), a SLV of ST-181 at the *adk* locus, first encountered in Niger in 2006. All these isolates were serogroup X. Of the 27 ST-181 complex isolates analyzed for PorA and FetA, all were P1.5-1,10-1, while FetA varied with 74% F1–31, 22% F4–23, and 4% F5–69. All 48 ST-181 complex isolates originated from West Africa: Niger (n = 35), Burkina Faso (n = 10), Benin (n = 1) and Togo (n = 2).

Serogroup X causing disease in East Africa (Kenya and Uganda) belonged to ST-5403, a clone unrelated to the ST-181 complex and not yet assigned to a clonal complex. Of the 15 ST-5403 isolates that were sequenced for *porA* and *fetA* genes, 14 were P1.19,26/F3–27; one was P1.19,26-4/F3–27.

The serogroup Y isolates were mostly represented by the ST-23 and ST-167 clonal complexes. The ST-23 complex comprised 11 isolates of ST-4375 originating from Burkina Faso (n = 10) and Niger (n = 1), as well as one ST-2954 isolate from Benin. The ST-167 complex was represented by nine ST-767 isolates from Benin (n = 2) and Mali (n = 7), one ST-2880 isolate from Burkina Faso and one ST-8620 isolates from Mali. Both ST-2880 and ST-8620 are SLVs of ST-767, differing in the *aroE* and *fumC* loci, respectively. Ten of the ST-167 complex isolates were P1.5-1,10-8/F1–3 and one was P1.5-1,10-8/F3-1. All seven serogroup Y ST-4375 isolates analyzed were P1.5-1,2-2/F5–8.

### Temporal changes within countries

**Figure 1:**
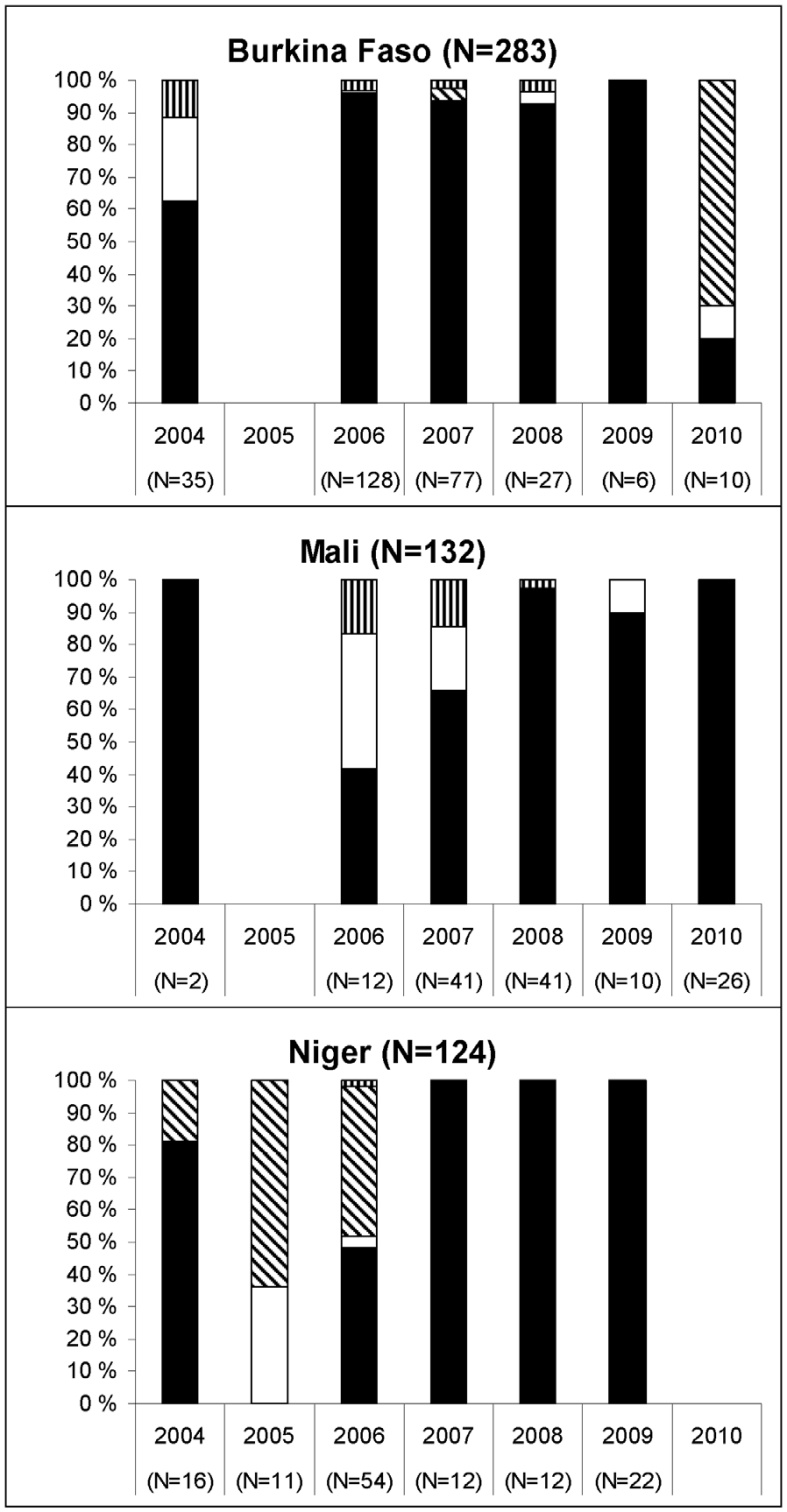
Serogroup distribution (%) in Burkina Faso, Mali and Niger in the period 2004–2010. In the figure, the few non-groupable isolates were included together with those assigned to a serogroup on the basis of the capsule PCR and/or their molecular profile, except for ST-192 isolates (n = 2) that were likely to harbour a capsule null gene [36]. Black bars: serogroup A; white bars: serogroup W135; oblique strips: serogroup X; vertical strips: serogroup Y.

The disease-causing strains varied in individual countries of the meningitis belt within the reported time interval. For the three countries with most analyzed isolates, the serogroup distribution by year is shown in Fig. 1. In Burkina Faso, serogroup A dominated during the whole period except for 2010, when serogroups X, A and W135 were represented by 7, 2 and 1 strains, respectively. In Mali, serogroup A has been responsible for ∼80% of cases in all the years, but in 2006 the proportion of serogroup A and serogroup W135 isolates was equal. In Niger serogroup A isolates dominated except for 2005–2006, when serogroup X prevailed. Unfortunately, we did not obtain any case isolates from Niger in 2010. However, an increase in isolates of serogroup W135 and a decline in serogroup X were reported in Niger in 2010 [4].

In Chad, serogroup W135 dominated during the whole period except for 2007 and 2010, when serogroup A was prevalent; but the numbers of isolates were small.

### Evolution of ST-5 complex

**Figure 2:**
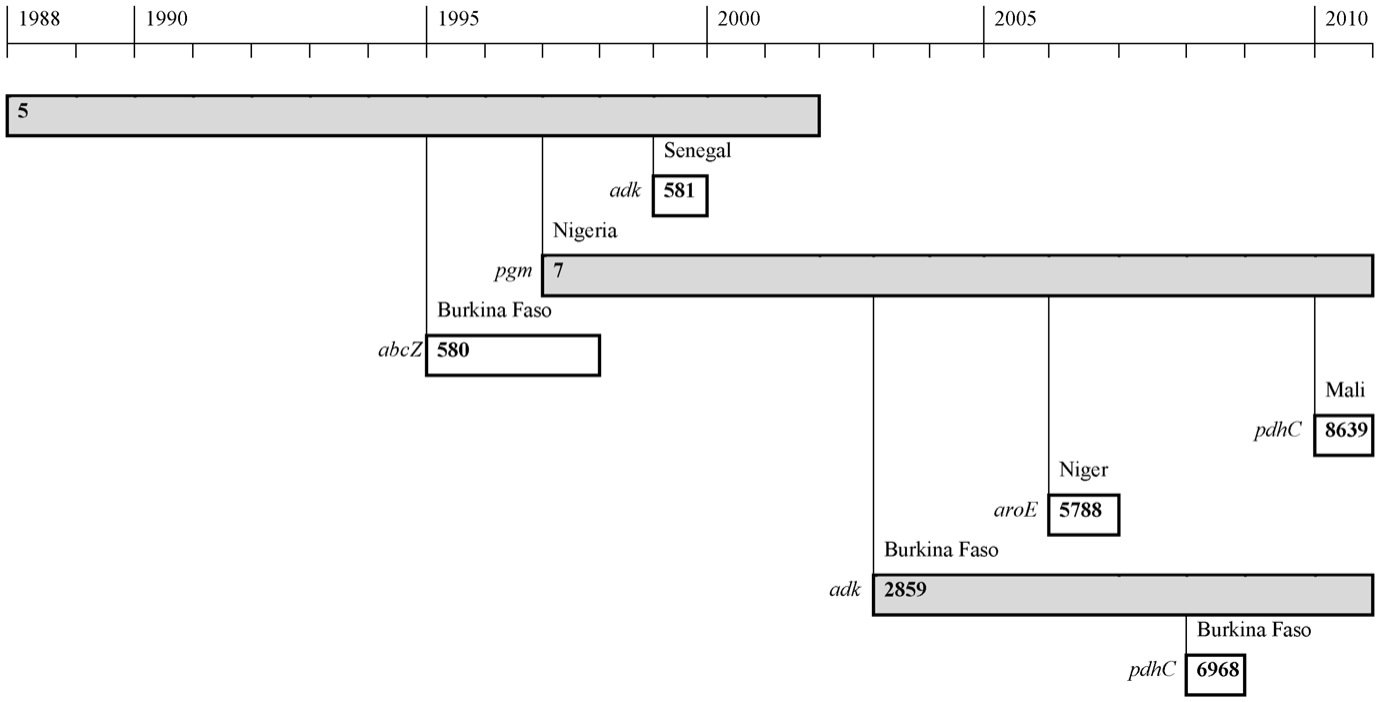
Evolution of the ST-5 complex in the meningitis belt in the period 1988–2010. The ST is shown within the bar, the country where the new ST was first detected is indicated above and the locus changed to the left. The left end of the bar shows when the variant was first detected and the right end shows the last reported isolation. The three dominant STs are marked with grey background.

Since the introduction of the ST-5 complex in the meningitis belt in 1987 [9], variation in housekeeping genes has occurred, resulting in new STs (Fig. 2). In the period 2004–2010, ST-7, which had already totally replaced ST-5 in the meningitis belt by 2003 [18], and ST-2859, which emerged in Burkina Faso in 2003, dominated within the ST-5 complex. In addition, three new STs emerged during the period 2004–2010: ST-6968, a single locus variant (SLV) of ST-2859 in the *pdhC* locus was first identified in Burkina Faso in 2008; ST-5788 and ST-8639 were first identified in Niger in 2006 and Mali in 2010, respectively, and are SLVs of ST-7, in the *aroE* and *pdhC* loci, respectively. In contrast, no variation in the genes coding for the surface exposed outer membrane proteins PorA and FetA has been detected.

## Discussion

This study included 773 isolates recovered from meningococcal disease cases in 13 countries of the meningitis belt during a 7-year period. Only 19 distinct STs were revealed. These STs belonged to 6 clonal complexes, two of which, the ST-5 and the ST-11 complexes, accounted for 83.6% of the isolates.

### Limitation of the study

In the period 2004 to 2010 approximately 225,000 meningitis cases from 12 countries within the African meningitis belt were reported by the WHO Inter country Support Team – West Africa, which compiles information from most countries of the sub-Saharan Africa [30]. This is summarized in Table 4 which showed a relatively quiet year in 2005 with less than 7,000 reported cases to the peak year of 2009 with nearly 80,000 reported cases. Of the reported meningitis cases less than 10% have a clinical specimen taken and examined microbiologically in the country of origin. Meningococcal meningitis was laboratory-confirmed for 7803 patients, i.e. about for one third of the patients from whom a clinical sample was analysed.

**Table 4:** Number (%) of studied isolates in relation to the number of laboratory-confirmed cases and number of reported cases per year in countries of the meningitis belt.

Year	No. of reported cases	No. of CSF samples analysed	No. of laboratory-confirmed MD cases	No. of isolates in the study (%)
2004	31,712	NA^a^	809	71 (8.8)
2005	9,876	1,193	190	17 (8.9)
2006	41,526	6,215	1,572	225 (14.3)
2007	45,997	2,533	680	183 (26.9)
2008	33,381	3,413	1,134	107 (9.4)
2009	88,199	5,688	2,210	92 (4.2)
2010	30,103	4,132	1,238	78 (6.3)

Data source WHO Inter country Support Team – WestAfrica [30]. Numbers from the Democratic Republic of Congo have been removed to include only countries of the meningitis belt.

a NA, Information not available.

We here report the molecular characterization of 773 isolates collected over this 7-year period. In our previous study, encompassing a period of 15 years, data on a total of 357 isolates had been collected [18]. This shows an overall improvement likely resulting from a better use of the laboratory in meningitis diagnosis in Africa and an increase networking of the WHO Collaborating Centers. However, in comparison to the huge number of cases occurring in the region, only a very small fraction of cases were represented in our analysis. Relating to the data collated by the WHO Inter country Support Team – West Africa the proportion of culture-confirmed meningococcal cases included in our study ranged from 4.2% in 2009 to 27.2% in 2007 (Table 4). The representation of the different countries was variable; especially we were unable to obtain strains from the Ivory Coast and Ethiopia. From these two countries, however, only 32 and 51 cases, respectively, have been culture-confirmed in this 7-year period according to reported data [30]. From the remaining countries of the meningitis belt that report to the WHO Inter country Support Team – West Africa, our collection included from 2.5% of the laboratory-confirmed cases from Niger (124 of 5061 cases) and up to 70.6% of the laboratory-confirmed cases from Mali (132 of 187 cases). These data illustrate the great differences both in surveillance systems and laboratory-based diagnosis of meningococcal meningitis between countries of the belt.

Comparing our results for the 3 countries with most isolates (Fig. 1) with the serogroup distribution reported in the WHO Meningitis Weekly Bulletin, we see however similar proportions and trends [30]. In Burkina Faso, 1,090 meningitis cases were laboratory-confirmed in-country for the 7-year period. The great majority of isolates were serogroup A, except for 2004 and 2010 when a mixed situation was seen. In 2004, the second serogroup was W135, while in 2010 serogroup X emerged toward the end of the season; these situations were evidenced both by the in-country surveillance and our strain collection.

Serogroup A dominated in Mali during the entire period. The mixed population we described in 2006 and 2007 was not evident from the WHO Meningitis Weekly Bulletin, where a few non-A cases were laboratory-confirmed (1 of 36 in 2006 and 5 of 60 in 2007) [30].

In Niger the National Reference Laboratory (CERMES) in Niamey provides a remarkable example of what kind of laboratory support can be achieved in West Africa. More than one thousand meningococcal meningitis cases have been confirmed and serogrouped during the most severe epidemic years of 2006 and 2009. The representativeness of the small fraction of isolates from Niger examined in this work is shown by the gradual increase in serogroup X until 2006, followed by a new wave of serogroup A cases in 2009.

For the other countries data are sporadic and our strain collection can only show which clonal complex and specific variant has been responsible for specific outbreaks.

### Dominance of serogroup A

Serogroup A *N. meningitidis* was responsible for most of the meningococcal outbreaks in 2004–2010, as it was for the period 1988–2003 [18]. The genetic diversity of serogroup A strains was low as all 568 isolates belonged to only five closely related STs of the ST-5 complex and all 344 isolates analyzed for PorA and FetA were P1.20,9/F3-1. Interestingly, this *porA-fetA* sequence combination has been stable among the serogroup A strains that cause disease in the meningitis belt for more than two decades, which suggests that the immunologic selection pressure on these outer membrane proteins is low. This is in contrast to the high variability identified in the genes coding for these two potential vaccine antigens among serogroup B isolates [31], [32]. Serogroup A strains of the ST-5 complex from other geographical areas may also harbour the P1.20,9/F3-1 antigen combination, but variants, especially for FetA, are not uncommon (http://pubmlst.org/neisseria/). This observation should be considered when developing strategies for protein-based vaccines for Africa.

Indeed, SLVs and double locus variants were detected among serogroup A isolates belonging to the ST-5 complex, and some of them (ST-7 and ST-2859) were clearly successful in successive clonal replacement within the complex. This study confirms complete replacement of ST-5 with ST-7 that occurred after 2001 [18], as none of the strains from the period 2004–2010 were ST-5. However, during this period ST-2859 which was first discovered in Burkina Faso in 2003 completely replaced ST-7 in that country. No serogroup A isolates recovered from Burkina Faso since 2003 were ST-7. The total replacement of ST-7 by ST-2859 in Burkina Faso was also evidenced in a carriage study performed in 2009 when ST-2859 was identified as the only serogroup A clone circulating among healthy carriers [28]. ST-2859 has spread from Burkina Faso to Mali in 2004 and to Niger in 2008. In these two countries, however, replacement of ST-7 by ST-2859 has not been complete, as both clones were still recovered in Niger in 2009 and in Mali in 2010. ST-2859 has not been observed in the other countries of the meningitis belt. The severe epidemic in Nigeria in 2009 (more than 56,000 reported cases) was caused by ST-7.

### Continuous spread of serogroup W135

The first large epidemic caused by serogroup W135, ST-11, occurred in Burkina Faso in 2002, probably following amplification of this clone after the annual Hajj pilgrimages of 2000 and 2001 [7], [33]. Prior to the Burkina Faso epidemic, serogroup W135 ST-11 isolates had been identified in Chad in 1996, Cameroon and Senegal in 2000, CAR in 2001, showing a large dispersion of the clone in the meningitis belt [18]. In the period 2004–2010, the ST-11 complex was identified in all the countries of the meningitis belt where isolates were obtained, except for Niger and Togo. While ST-11 complex was present in Niger in 2002 and 2003, from 2004 the W135 isolates recovered from that country belonged to ST-2881 of the ST-175 complex. In 2010, W135 predominated in Niger [30], but no isolates were available to us. Laboratory identification of the etiological agent was mainly performed by PCR, but all 9 W135 isolates tested by Collard and co-workers [4] belonged to ST-11. The ST-11 complex seemed to disappear also from Burkina Faso after 2006 and the few W135 recovered then belonged to the ST-175 complex. In Chad serogroup W135 dominated during most of the period 2004–2010, belonging either to ST-11 (2005, 2006, 2008–2010) or ST-2881 (2004 and 2007). In the years 2008–2010, the proportion of serogroup W135 in Chad decreased while the proportion of serogroup A increased (Fig. 1). In Niger, isolates of serogroup W135 belonging to ST-11 accounted for nearly 50% of the case isolates in 2010 [4]. In contrast to the ST-11 complex, the ST-175 complex has not been associated with major epidemics and is responsible for endemic disease cases. This clonal complex is geographically widespread and has been identified in meningococcal carriage studies in Europe and Asia (http://pubmlst.org/neisseria/).

### Emergence of serogroup X

Carriage and sporadic disease-associated serogroup X isolates have been detected in the meningitis belt as early as in the 1990s [6], [8], [34]. During the 7-year period analysed here, Niger experienced a serogroup X outbreak in 2006 [3] and the responsible epidemic strain was ST-181 (Table 1). Serogroup X, however, declined later on in Niger and represented less than 1% of the isolates in 2010 [4]. Delrieu and coworkers reported a serogroup X outbreak in one district in Togo in 2007 [35]. Although no isolates had been stored, MLST on cerebrospinal samples revealed ST-181 [35]. This serogroup X strain was present in Burkina Faso in a small fraction (4%) of the cases in 2007, but emerged as an important cause of disease in 2010 where it was identified in 7 of our 10 meningococcal isolates. Serogroup X outbreaks occurring in northern and central regions of Burkina Faso in 2010 have been documented [35]. In 2009, serogroup X ST-181 had been identified in 0.44% of healthy carriers in Burkina Faso [28], with a higher prevalence in the north-east district of Kaya, close to Niger (1.05%). The circulation of serogroup X meningococci among asymptomatic carriers and the high incidence of serogroup X disease before MenAfriVac introduction should be kept in mind when evaluating possible serogroup replacement following use of the vaccine. The possible spread of serogroup X has to be closely monitored in the coming years as there are yet no available vaccines against this serogroup.

Interestingly, serogroup X meningococcal disease also emerged in Kenya and in Uganda in 2006, but these cases were caused by a strain unrelated to the ST-181 complex [8].

### Conclusions

Our data show that before introduction of MenAfriVac in the African meningitis belt, serogroup A *N. meningitidis* of the ST-5 complex remains the predominant disease-causing clonal complex, with two STs (ST-7 and ST-2859) contributing to severe outbreaks in different countries. The situation, however, is heterogeneous both temporally and geographically. After the emergence of serogroup X ST-181 complex in Niger in 2006, the clone became a significant cause of disease in Burkina Faso in 2010 before vaccine introduction. Improved laboratory confirmation of meningitis cases is essential to assess changes in the disease epidemiology. Culture of the disease-causing organism and their genotyping are necessary to a better understanding of the bacterial population dynamics.
